# The road forward to incorporate seawater microbes in predictive reef monitoring

**DOI:** 10.1186/s40793-023-00543-4

**Published:** 2024-01-15

**Authors:** Marko Terzin, Patrick W. Laffy, Steven Robbins, Yun Kit Yeoh, Pedro R. Frade, Bettina Glasl, Nicole S. Webster, David G. Bourne

**Affiliations:** 1 Australian Institute of Marine Science, PMB no3 Townsville MC, Townsville, QLD 4810 Australia; 2 College of Science and Engineering, James Cook University, Townsville, QLD 4811 Australia; 3 AIMS@JCU, James Cook University, Townsville, QLD 4811 Australia; 4 Australian Centre for Ecogenomics, University of Queensland, St. Lucia, QLD 4072 Australia; 5 Australian Antarctic Program, Department of Climate Change, Energy, the Environment and Water, Kingston, TAS 7050 Australia; 6 Natural History Museum Vienna, 1010 Vienna, Austria; 7 Division of Microbial Ecology, Centre for Microbiology and Environmental Systems Science, University of Vienna, 1030 Vienna, Austria

**Keywords:** Microbial monitoring, Reef bacterioplankton, Functional meta-omics, Coral reef health

## Abstract

Marine bacterioplankton underpin the health and function of coral reefs and respond in a rapid and sensitive manner to environmental changes that affect reef ecosystem stability. Numerous meta-omics surveys over recent years have documented persistent associations of opportunistic seawater microbial taxa, and their associated functions, with metrics of environmental stress and poor reef health (e.g. elevated temperature, nutrient loads and macroalgae cover). Through positive feedback mechanisms, disturbance-triggered heterotrophic activity of seawater microbes is hypothesised to drive keystone benthic organisms towards the limit of their resilience and translate into shifts in biogeochemical cycles which influence marine food webs, ultimately affecting entire reef ecosystems. However, despite nearly two decades of work in this space, a major limitation to using seawater microbes in reef monitoring is a lack of a unified and focused approach that would move beyond the indicator discovery phase and towards the development of rapid microbial indicator assays for (near) real-time reef management and decision-making. By reviewing the current state of knowledge, we provide a comprehensive framework (defined as five phases of research and innovation) to catalyse a shift from fundamental to applied research, allowing us to move from descriptive to predictive reef monitoring, and from reactive to proactive reef management.

## Introduction

Coral reefs are some of the most biodiverse and productive aquatic environments on the planet, providing shelter, nutrition, and habitat for many marine species, and offering valuable ecosystem services to humans, including protection of coastal areas, tourism, and fisheries [1, 2]. Despite their ecological significance and economic value, coral reefs have suffered major declines in recent decades due to the synergistic effects of local chronic impacts and global climate change [3, 4] with recent estimates indicating that half the world’s coral cover has been lost since the 1950s [5]. To preserve coral reefs, an improved understanding is needed of the mechanisms involved in coral resilience to local and global environmental stressors.

Marine microorganisms account for ~ 65–90% of the marine biomass [6, 7] and therefore constitute the life support system of the biosphere, being central to planetary marine food webs and biogeochemical cycles, and responsible for approximately 50% of the world’s primary production [7–14]. Marine plankton also play a vital role in the stability and function of coral reefs by providing crucial ecosystem services. Heterotrophic microbes in reef seawater rapidly capture and recycle nutrients from the water column, for example, dissolving coral derived mucus before it sinks to the sediment [15]. By rapidly taking up nutrients from the water column, seawater microbes have a critical role in making these nutrients available to higher trophic levels [15–18]. This efficient recycling of nutrients ultimately allows corals to thrive in oligotrophic and nutrient-deplete environments, often referred to as ‘marine deserts’ [18].

Host-associated microbes also provide various functions to their metazoan hosts, including nutrition, removal of waste products (e.g. ammonia), protection from invading pathogens, and stimulation of developmental processes and morphogenesis [19–24]. However, environmental stressors such as eutrophication and elevated temperatures may shift host-associated microbial communities from mutualistic to pathogenic states once critical thresholds are reached [25–27]. The emergence of copiotrophic and potentially pathogenic microbes (e.g. *Flavobacteriaceae*, *Cryomorphaceae*, *Rhodobacteraceae*, *Rhodospirillaceae*, *Vibrio*) along with their associated functions (e.g. virulence factors and toxin production) has been associated with increases in coral diseases leading to tissue necrosis, and ultimately partial or whole colony mortality [26, 28, 29].

This sensitivity of reef microbes to environmental perturbations potentially allows microbes to be used as indicators of environmental change in the surrounding reef [30, 31]. Importantly, reef microorganisms may represent early warning indicators of environmental disturbances since microbial communities change in their composition and function before the development of visual signs of stress, such as coral disease, bleaching, and tissue necrosis [25, 30–39]. These traditional visual signs of reef disturbance often become evident only after prolonged periods and potentially once ecosystem tipping points are reached [40]. Current monitoring efforts are therefore often reactive, reporting the outcomes of impact with limited potential to mitigate future reef decline. Incorporating microbial processes within reef monitoring frameworks could represent a powerful way to observe early signs of stress, providing more time to implement reef management strategies and mitigate the impacts of environmental disturbances on reefs [30, 31].

**Fig. 1:**
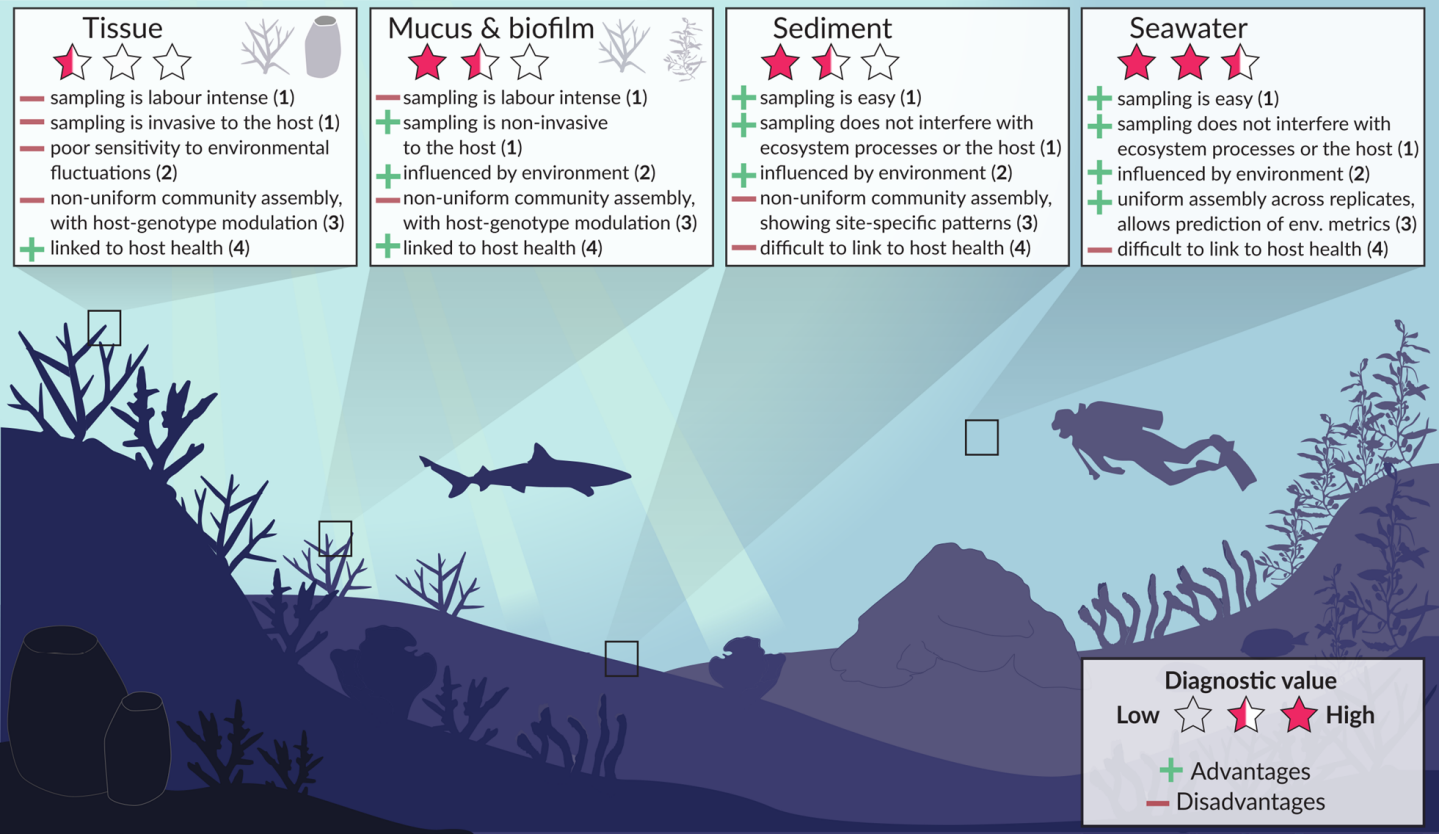
Overview of the diagnostic value of various coral reef microbiomes. The diagnostic value (indicated as stars) is based on the sum of advantages (+) and disadvantages (−) for key characteristics of optimal microbial indicators: (1) ease of sampling, (2) sensitivity towards environmental fluctuations, (3) uniformity of community assembly, (4) our ability to link microbiome shifts to host health. Based on these criteria, seawater microbial communities collectively have the highest diagnostic potential to be used as microbial indicators of reef health, followed by sediment-associated and host-associated microbial communities, respectively. Free-living microbial communities (seawater and sediment) can be easily collected, without interfering with ecosystem processes and/or the health of reef organisms, consistent with desirable characteristics for environmental monitoring programs. In contrast, the collection of host-associated microbiomes is labour intensive and potentially poses a certain risk for host health when collecting tissue, although collections of the host-biofilm are non-invasive for the host. Seawater also revealed the highest sensitivity to changes in the surrounding environment (e.g., temperature and eutrophication) due to uniform community assembly patterns of the seawater microbiome across replicates, while sediments were primarily influenced by site-specific patterns (e.g. grain size) and host-associated microbiomes predominantly showed a host-genotype modulation. While the diagnostic value is highest for most criteria in the seawater microbiome, it is challenging to link disturbance-induced shifts in marine bacterioplankton to host health. Given the importance of host-associated microbes to the health of reef holobionts, the establishment of microbial baselines for host-associated microbiomes and the search for host health microbial indicators are still warranted

A framework to implement microbial observations within reef monitoring programs was recently proposed [36]. Using indicator value analysis and machine learning approaches, seawater microbial communities (inferred from 16S rRNA gene amplicon sequencing data) were documented to provide accurate predictions of water temperature and eutrophication states of reefs. In contrast, macroalgae, coral and sponge microbiomes were predominantly structured by the host organism and less influenced by the environment [36]. Exposure of coral and sponge species to non-lethal stressors (temperature, acidification and salinity) in controlled experimental systems has similarly demonstrated that host factors strongly influence host-associated microbiomes [41–45], possibly limiting their use as early indicators of stress in reef monitoring. These trends were recently also documented at scale, using 16S rRNA gene amplicon sequencing data from the Tara Pacific Expedition [46]. Only 4–11% of variance in the coral microbiomes surveyed (*Millepora*, *Porites* and *Pocillopora*) was explained by physicochemical properties of the seawater, compared with ~ 30% variance in planktonic microbial communities explained by water chemistry [46]. Considering that seawater microbes provide accurate diagnostics of temperature and eutrophication states in the reef environment [36] and that seawater can be easily collected alongside in situ reef health surveys in a cost-effective and non-destructive manner, we assert there is realistic scope to incorporate microbial observations of seawater microbes alongside ongoing in situ reef health surveys (Fig. 1).

**Fig. 2:**
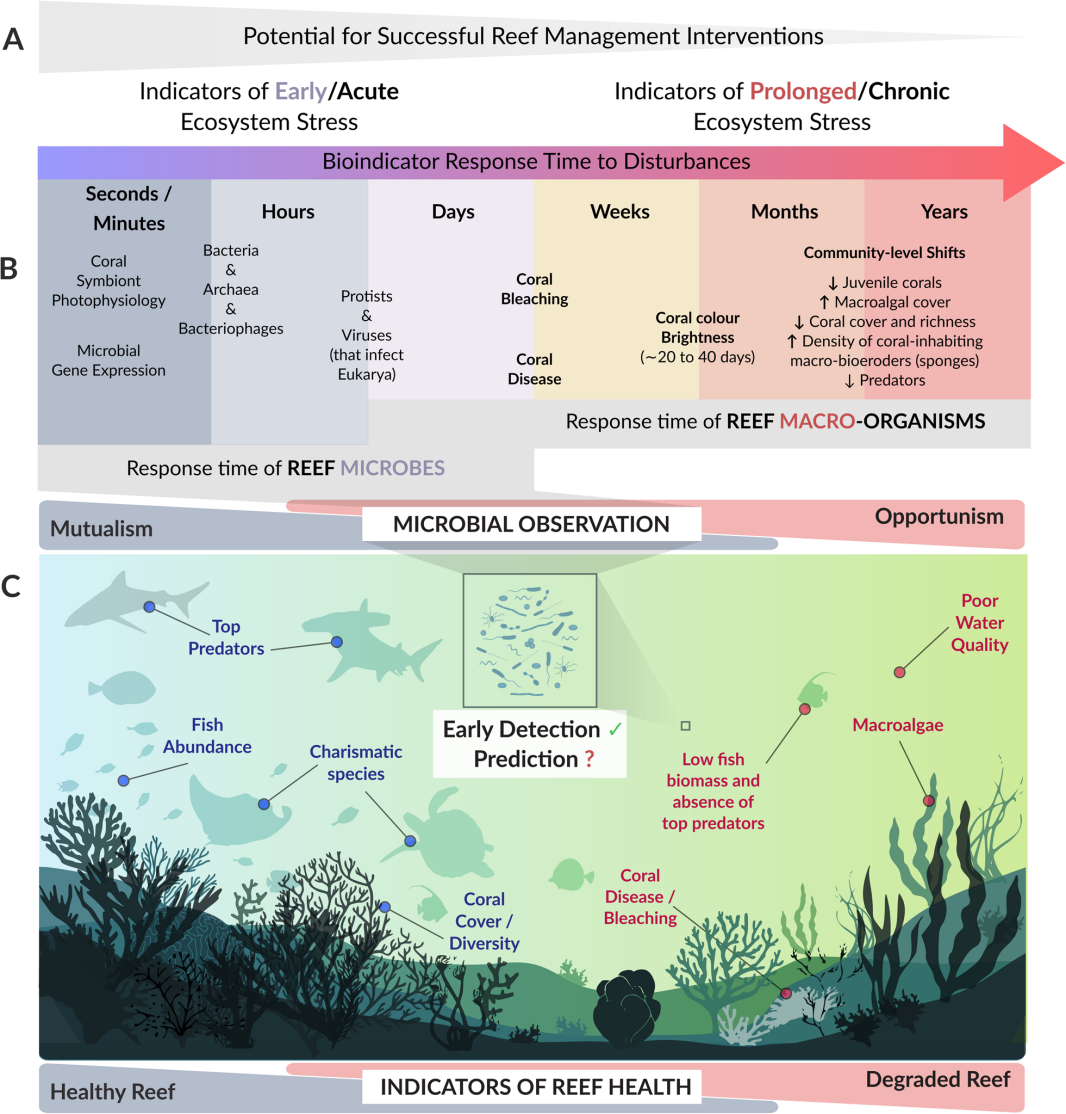
Potential of reef seawater microbes to inform on reef health status. Successful reef management interventions need to rely on acute and early identification of changes in the reef, before ecosystem ‘tipping points’ are reached (**A**). However, most reef monitoring programs are based on visual signs to assess ecosystem stress (e.g., coral disease, bleaching and community-level shifts), which become evident only after prolonged environmental disturbances (**B**). Due to their short generation times, seawater microbes respond rapidly to environmental changes, and it has therefore been well established that marine bacterioplankton allows accurate and early diagnostics of environmental fluctuations in the reef (**C**, middle). However, the predictive potential of the seawater microbiome has been largely unexplored and it remains unclear how environmental changes will alter microbial functioning of reef bacterioplankton, and how this may translate to reef ecosystem functioning via cascading effects and feedback loops (**C**, middle). Figure 2C was adjusted from Vanwonterghem and Webster [18] with permission from authors

**Fig. 3:**
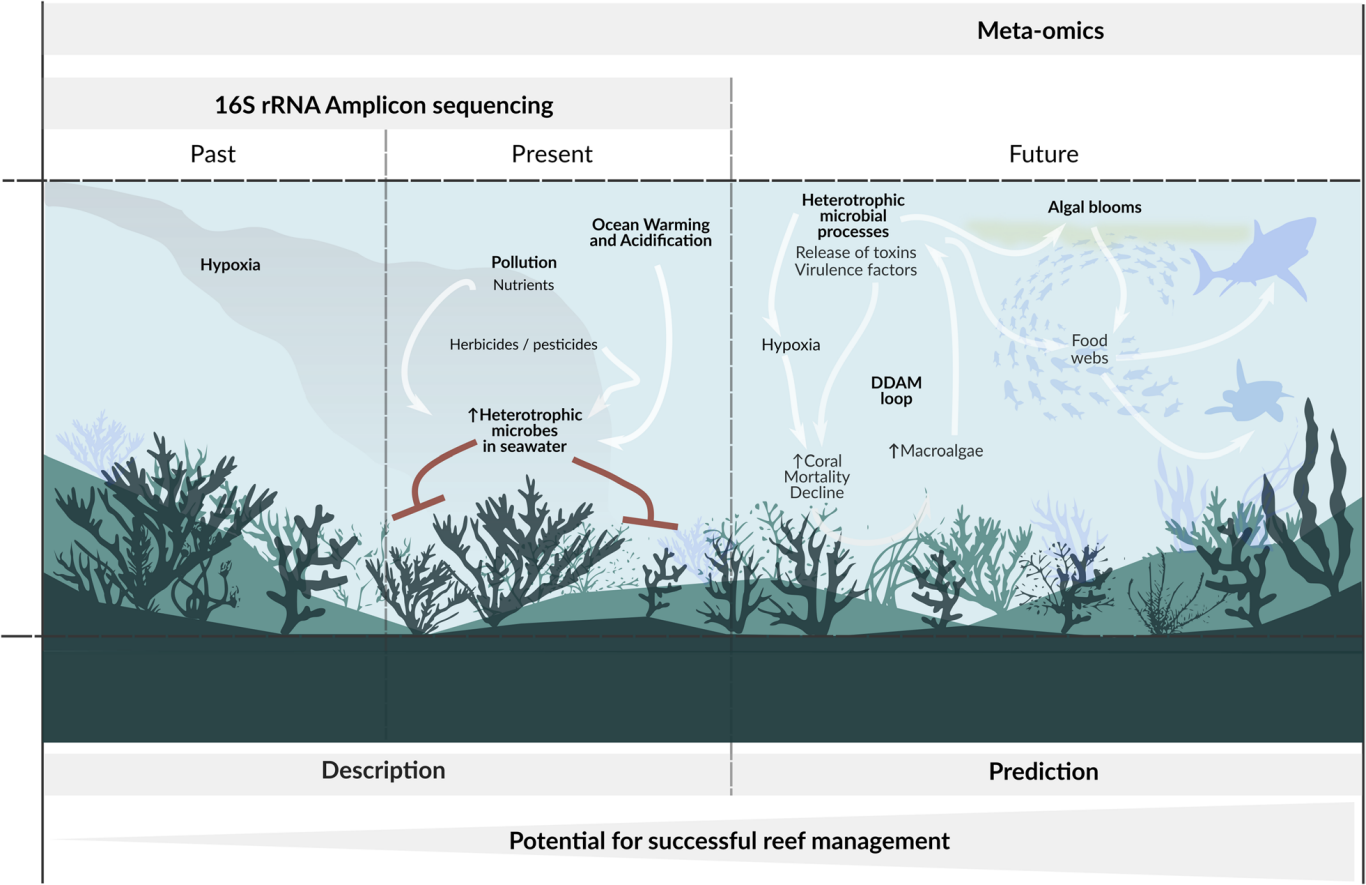
Reef microbial observation should extend beyond taxonomy and towards function, to move from descriptive to predictive reef monitoring. 16S rRNA amplicon-sequence data has clearly shown that opportunistic and potentially pathogenic microbes robustly correlate to degraded reefs characterised by poor water quality, increased macroalgae cover and coral disease/bleaching. However, amplicon-sequence data has a limited resolution to go beyond description of past or present changes in the reef, as the consequences of the enrichment of particular microbial indicator taxa on reef health often cannot be inferred from microbial taxonomy alone (left, shown in red). Microbial meta-omics data would allow prediction of how environmental changes will affect the services microbes provide to coral reefs (e.g., primary productivity, nutrient/biogeochemical cycling, and exposure to pathogens), and how the altered microbial activity may translate to reef ecosystem dynamics (right). This predictive monitoring is needed for successful reef management and decision making (bottom)

Apart from early detection of environmental changes (Fig. 2), seawater microbes are also important to predict reef functioning as environmental perturbations can destabilise reef bacterioplankton and alter their ecosystem services (Fig. 3), resulting in adverse implications on future reef dynamics via cascading effects and feedback loops [39, 47, 48]. For example, cumulative effects of nutrient eutrophication and elevated temperature can trigger heterotrophic microbial activity in seawater, resulting in harmful algae blooms and hypoxia at reef scales causing rapid coral mortality [48–50]. Heterotrophic seawater microbes were also proposed to be central to large-scale reef declines caused by chronic stressors such as elevated nutrients and overfishing via the DDAM (disease, dissolved organic carbon (DOC), algae and microbes) model [47, 51, 52]. The DDAM positive feedback loop begins with eutrophication and overfishing facilitating growth of fleshy macroalgae, which confers a competitive advantage to other macroalgae over coralline algae and calcifying corals by preventing settlement of coral larvae [51]. At the same time, ocean warming caused by climate change stimulates the release of dissolved organic carbon (DOC) by fleshy macroalgae, which results in the proliferation of copiotrophic and potentially pathogenic bacterial communities in seawater, a process referred to as microbialisation. Increased abundance and activity of opportunistic and potentially pathogenic microbes in the water column further fuels the DDAM positive feedback loop by causing additional coral decline through increased coral disease prevalence, which ultimately maintains algal competitive dominance [51]. This concept of microbialisation links changes in seawater reef microbes to reef health decline and is therefore important from a predictive monitoring perspective (Fig. 3).

Currently most of what we know about the potential for seawater microbes to predict reef health has been inferred from microbial taxonomy rather than microbial function [18]. Amplicon sequencing of the 16S rRNA gene (the universal taxonomic marker gene in bacteria and archaea) has identified opportunistic and potentially pathogenic microbes persistently associated with degraded reefs across independent meta-omics studies [26, 39, 52–55]. However, it is still unclear how seawater microbes can be applied as indicators of coral reef ecosystem health as (1) functional characterisation (i.e. survey of functional potential via metagenomic sequencing) of reef bacterioplankton is still largely lacking (but see Tara Pacific Expedition, [56–58, 65]), and because (2) frameworks still need to resolve the ecologically important functions that seawater microorganisms provide to the reef ecosystem. Meta-omics approaches (e.g., metagenomics, metatranscriptomics, metaproteomics and metabolomics) that survey specific functional genes extend beyond taxonomy and would allow scalable investigation of adaptive (i.e. community turnover) and acclimatory (e.g. physiological and gene expression changes) responses of microbes to their environment [59]. For example, undertaking meta-omic surveys to document microbial functional potential as part of reef microbial observations can elucidate how environmental change affects ecosystem services that seawater microbes provide to coral reefs, and to predict how this may translate to future reef dynamics (Fig. 3). However, when applying meta-omics for ecosystem monitoring, there are numerous strengths and weaknesses associated with the various technologies that need to be considered. These methodological considerations have been extensively reviewed previously [59–64] and are therefore not covered in detail here.

**Fig. 4:**
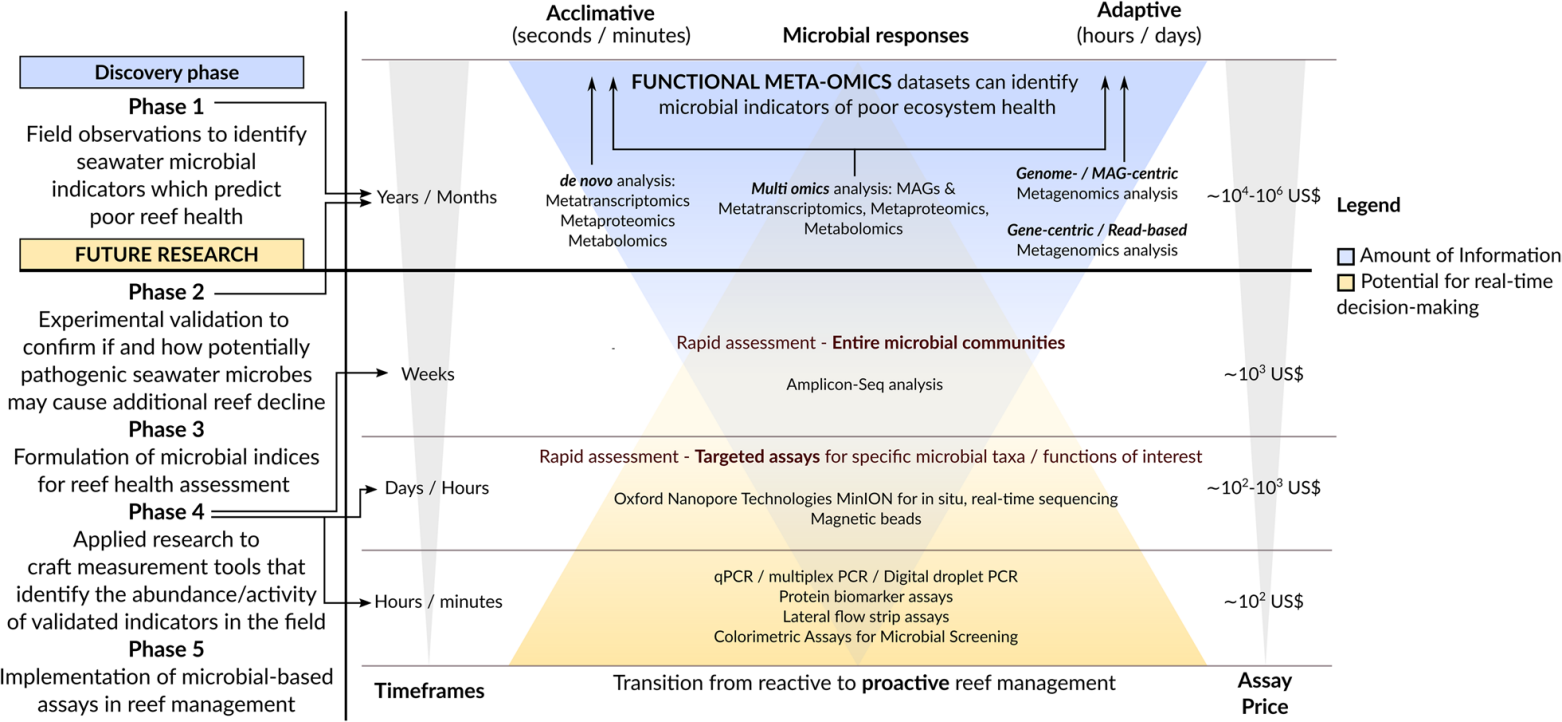
The proposed five-step framework of research and innovation to move from descriptive to predictive reef microbial monitoring, and from reactive to proactive reef management. Functional meta-omics datasets are critical to discover microbial indicators of poor reef health in the field (Phase 1), however high costs (see ‘Assay price’) and long bioinformatics processing times (see ‘Timeframes’) of microbial meta-omics datasets suggest their limited utility for rapid decision-making in reef management. We highlight that this milestone has been largely achieved through various localised studies, though in the years to come, the integration of recently generated datasets obtained in large-scale surveys (most notably the Tara Pacific Expedition) will be crucial to understand the ubiquity of identified microbial indicators at global scales. Once microbial indicators of poor ecosystem health are identified based on functional meta-omics datasets, experimental validation (Phase 2) is needed to confirm the same patterns occur in laboratory conditions, as well as to identify the causality of microbiome-environment associations from the field, which we predict still remains a distant goal and will require years of research. Once experimentally validated, microbial indices can be formulated (Phase 3) and applied research can commence to develop rapid (within weeks, days or minutes, see ‘Timeframes’) and cost-effective (see ‘Assay price’) assays to quickly assess reef health in the field (Phase 4), which can be used in proactive reef management and rapid decision-making (Phase 5)

In this review, we demonstrate that there is realistic scope to extend reef monitoring efforts by including microbial meta-omic surveys that capture the metabolic and functional potential of free-living microbes in seawater. We discuss the current state of using seawater microbial indicators in reef monitoring programs in the context of a five-step framework (Fig. 4). We hope this review will accelerate the shift from fundamental towards applied research to develop rapid and cost-effective microbial-based assays for assessment of reef health, which would be invaluable in predictive reef monitoring and proactive management.

## Seawater microbes are essential to predict ocean and reef health

Enormous amounts of molecular and environmental data have been collected in recent years on oceanic microbes, both through various global sampling efforts that collected snapshots of marine microbes in time and space [8, 10–12, 14, 56–58, 65–67] as well as various long-term microbial observatory stations [13]. These large microbial oceanography initiatives aim to predict how environmental change alters the distribution patterns as well as taxonomic and functional diversity of ocean plankton at global scales [7, 9, 14, 68, 69]. To successfully integrate these large and complex datasets, novel computational approaches such as multi-omics data integration [70], ecological niche modelling [71, 72], network analysis [72, 73], multivariate statistics and supervised learning needed to be applied, and these modelling and data integration efforts have already enabled marine scientists to transition from hindcasting to forecasting. For example, tropical marine biogeographical provinces are predicted to expand towards the poles due to climate change (at the expense of temperate and polar zones), followed by a compositional shift in marine plankton which is projected to decrease carbon export fluxes and affect nitrogen cycling [69]. Furthermore, marine viruses were identified as the best predictors of global ocean carbon flux in comparison with archaea, bacteria and eukaryotes [73]. Further integration of seawater microbial meta-omics data into models of Earth system functioning will be crucial to improve such models, as marine bacterioplankton are directly involved in the processes being modelled such as biogeochemical cycling, primary production, and carbon efflux under climate change scenarios [74].

Statistical learning models have also been applied to coral reefs to identify microbial indicators that inform ecosystem health, though at comparatively smaller scales [36, 39]. For example, random forest machine learning identified that seawater surface temperature in the Great Barrier Reef can be accurately predicted from reef bacterioplankton community structure [36], and a linear discriminant analysis (LDA) model was developed that accurately predicts reef categories (e.g. inshore, mid-shelf or offshore) in the GBR based on seawater microbial community profiles [39]. Importantly, vast amounts of meta-omic and multi-omic data streams have recently been collected on free-living and host-associated reef microbes in the Pacific Ocean (e.g. the Tara Pacific Expedition), which will allow further development of models to incorporate seawater microbes in predicting how climate change and human impact may affect reef functioning and health [56–58, 65, 75]. The Tara Pacific Expedition (2016–2018) has sampled seawater and coral for multi-omics sequencing in 32 island systems throughout the Pacific along with extensive environmental metadata, hence establishing spatial baselines of reef microbes in the Pacific Ocean [46, 56–58, 65, 75].

These large-scale meta- and multi-omics datasets will, for the first time, provide the necessary basis to assess how functions of reef seawater microbes (e.g. photosynthesis, nitrification, ammonia oxidation, sulfate reduction, methanogenesis, virulence etc.) shift with the environment at global scales. Such datasets will be crucial to extend beyond localised studies that have already identified seawater microbes indicating poor reef health, by establishing a robust baseline of microbial indicators that are shared across wide spatial and temporal scales. A recent literature review provided such a baseline, summarising how reef habitat degradation across regions in the Pacific Ocean and the Caribbean may alter microbe–DOM interactions, and the potential implications of shifts in microbial functioning contributing to further reef declines [76]. Further, analysis of large-scale meta-omics data of the seawater microbiome surrounding corals could provide insight about how local settings affect reef bacterioplankton, and how this may structure and affect dynamics of coral microbiomes [65, 75], improving our understanding of the role of seawater microbes in reef resilience and acclimatisation (Fig. 3). Considering the implication of free-living seawater microbes in feedback loops and cascading effects, developing regulatory guidelines is needed to protect seawater microbial functions that are ecologically relevant to the reef, which would be invaluable in reef monitoring if early detection of how specific microbial functions are disrupted could be used to predict and avoid additional coral mortality and reef declines [77].

## Experimental validation of microbes that predict poor reef health

Large meta- and multi-omic data streams such as Tara Pacific will be vital for identifying the indicator taxa and genes that predict poor reef health, however, this large data collection exercise is just one step in incorporating microbial related processes in reef monitoring frameworks (Fig. 4, phase 1). In many instances the microbial features selected by the models may be unrelated to degraded ecosystem health despite high correlations identified by the model [78]. As an example, an environmental overlap may exist between microbial indicators that correlate to coral bleaching (which occurs under accumulated thermal stress) and thermophilic bacteria which also proliferate at elevated temperatures, but do not impact reef health. Further, anaerobic bacteria and genes (which could indicate hypoxia in the reef) can still be present in the seawater days after a hypoxia-induced coral mortality event, and despite concentrations of dissolved oxygen having reverted to normal values [79]. Various additional factors may contribute to the noisy signal of microbial taxa-environment associations, including biotic interactions, limits to spatial dispersal and neutral demographic drift [80]. As there is a strong likelihood that only some microbial predictors of degraded reefs identified by the model will have a causal association with metrics of poor reef health, confirmatory experiments are needed to validate microbiome-environment associations [78] allowing interpretation of the suitability of the models and usefulness of the microbial indicator(s) (Fig. 4, phase 2), however this remains a distant goal. For example, while pathogenic microbes are hypothesised to play a role in coral disease, the causative microbial agents of coral disease have only been identified in a few cases, and often Koch postulates prove inconclusive [81–85]. The utility of seawater microbes as indicators of poor reef health should not be dismissed even when causal relationships are unknown, as there are promising microbes (most notably *Flavobacteriaceae*-affiliated taxa) that are persistently diagnostic of reef ecosystem degradation across independent omics studies [36, 39, 52–55, 86, 87]. Below we provide a shortlist of seawater taxa/genes that have been experimentally validated as indicators of poor reef health, and highlight they should be further studied to provide richer insights into their potential (causal) role in reef degradation.

Experimental validation of the DDAM loop and the microbialisation concept has confirmed bacterioplankton communities of coral- and algae-dominated reefs persist in laboratory conditions. A bottle experiment identified that rates of bacterioplankton growth and utilisation of DOC were elevated in algal exudate treatments compared to incubations with coral exudates and the control treatment, with macroalgae-derived sugars selecting for a less diverse community enriched in lineages of opportunistic *Gammaproteobacteria* including putative pathogens with known virulence factors (*Pseudoalteromonadaceae* and *Vibrionaceae*) [88]. Another bottle experiment identified that high DOC concentrations (as observed in macroalgae-enriched reefs) correlated to an enrichment of bacterial genera *Alteromonas*, *Oceanicola*, *Erythrobacter*, and *Alcanivorax*, which shifted in their metabolic capabilities from mutualistic towards pathogenic states via up-regulation of genes encoding for metalloproteases, siderophores, toxins, and antibiotic resistance factors [89]. Similar trends were identified by in situ mesocosm studies which placed benthic chambers over coral-, sand-, and macroalgae-dominated communities, to identify that macroalgae exudates facilitated a shift to a net heterotrophic system, with pelagic microbial communities displaying elevated consumption of macroalgae-released DOC as well as increased oxygen consumption [52]. Experimental validation of the DDAM loop clearly shows that the addition of macroalgae-derived nutrients (under laboratory conditions) causes microbial proliferation and a shift towards pathogenesis and carbon metabolism pathways that are less energetically efficient [52, 88, 89], as also observed in the field [47]. However, the cause-and-effect understanding of the DDAM mechanism still needs to be teased apart to understand if and how the increased abundance and activity of microbial copiotrophs and putative pathogens in seawater at macroalgae-enriched reefs directly contribute to coral disease and reef declines, before applying the concept of microbialisation in predictive reef monitoring and proactive management.

Hypoxia also represents a crucial mechanism in the DDAM feedback loop, as macroalgae-released DOC fuels heterotophic activity and respiration by seawater microbes, which can create localised hypoxic regions at the coral-macroalgae interaction zones [47, 76, 90–92]. Experimental studies show that the addition of antibiotics may eliminate hypoxia in coral–algal interfaces [90–92]. As hypoxia predominantly occurs at coral–algal interaction zones, reef water away from the benthos may not be enriched in anaerobic microbial taxa and genes (but see [93]). We therefore propose that monitoring for anaerobic microbes and functions is particularly relevant at the benthic and pelagic boundary layer, which represents a potential valuable environmental niche for reef monitoring to predict rapid declines in benthic organisms caused by hypoxia.

Another forecasting potential of seawater microbial monitoring is to predict coral disease outbreaks. The concept that animal disease outbreaks are driven by environmental change (e.g. climate warming) is well accepted across many terrestrial, freshwater and marine ecosystems [85, 94–98], and acquisition of microbial pathogens from the environment has been documented in some food- and waterborne diseases [37, 99]. For corals, there are many examples of putative microbial pathogens (isolated from diseased coral tissues) also being identified in the surrounding reef seawater, which often increase in their abundance and activity at elevated seawater temperatures [86, 100, 101]. The bacterial genus *Vibrio* sp. is particularly prominent as a potential causative agent of coral disease [84, 85]. A survey to elucidate *Vibrio* diversity in surrounding reef seawater using the well-curated pyrH (uridylate kinase) gene sequence identified that putative coral pathogens (i.e. *V. coralliilyticus, V. neptunis*, and *V. owensii*) were persistently present in the seawater of the Ishigaki coral reef system (Japan) across the entire 3-year survey period, with increased abundances correlated with elevated seawater temperatures for the majority of *Vibrio* species [86]. Another study identified a significant enrichment in *Planctomycetota* (lineages OM190 and CL500-3) and bacteria within genera *Synechococcus* and *Vibrio* during the marine heatwave on the GBR in April 2016, alongside an enrichment of *Vibrio*-derived virulence factors (i.e. metalloprotease genes vcpA, vcpB and vchA in *V. coralliilyticus*) [101]. Though as the authors highlight, a link between large-scale changes observed in the plankton-associated microbial community and reef ecosystem health could not be established based on their observations [101], which warrants for robust experimental validation to gain a cause-and-effect understanding between the presence of potentially pathogenic water-born microorganisms and coral disease outbreaks.

Interestingly, the signal of copiotrophic and potentially pathogenic microbes in seawater often persists even after the environmental disturbances have passed [79, 101], and this concept is known as the microbial ‘legacy effect’ [78]. Anaerobic microbes can persist in seawater for days after dissolved oxygen concentrations revert to normoxic values [79], and the microbial signal of potentially pathogenic microbes (e.g. *Planctomycetota* and *Vibrio*) and functions (e.g. *Vibrio*-derived metalloproteases) that were enriched during the marine heatwave on the GBR in April 2016 remained apparent until August 2016, months after the marine heatwave had dissipated [101]. This microbial ‘legacy’ effect may be important to explore from a monitoring perspective of free-living seawater microbes, as a shift in microbial functioning may cause additional coral decline even after the disturbance has passed. As an example, a number of studies have documented that coral disease outbreaks can exacerbate the impacts of bleaching events [102–107]. The cumulative stress corals face during thermal stress may make them susceptible to opportunistic microbial pathogens that persist in the seawater following marine heatwaves [101], further compromising their health and increasing mortality. Experimentally validating this ‘legacy effect’ may be crucial from a monitoring perspective to understand how long opportunistic microbes and functions persists after different environmental disturbances, and to predict if this may affect future reef dynamics.

## Formulation of seawater microbial indices for reef monitoring

Once experimentally validated, a list of microbial taxa and/or functions can be compiled to formulate robust microbial indicators which associate to metrics of poor reef health across both field and laboratory studies (Fig. 4, phase 3). Such efforts have already been made to formulate microbial indices for reef monitoring based on free-living seawater microbes. Most notably, microbialisation scores (defined as the ratio of metabolic rates between bacterioplankton and reef fish) have been proposed within the DDAM model as a metric of human impact on coral reefs [47, 76, 93, 108, 109]. This concept of microbialisation (a shift in biomass production and metabolic rates from macro to micro-organisms) has been well documented in macroalgae-enriched reefs in field observations [32, 109], across local and regional scales [47], and also validated experimentally in laboratory bottle experiments [88, 89, 110] and in situ mesocosm studies [52]. Despite their potential, microbialisation scores have not been implemented into standard reef monitoring efforts to date, primarily since the scores represent a metric relevant to shifting coral-algal dynamics, which is not universally applicable to reefs under environmental pressures that still maintain high coral cover and/or high fish biomass.

A recent meta-analysis of reef bacterioplankton identified several microbial indices of poor reef health across the Great Barrier Reef [39]. It was proposed that *Prochlorococcaceae* and *Synechococcaceae* families represent potential indicators of cross-shelf nutrient levels with an increasing *Synechococcaceae*:*Prochlorococcaceae* abundance ratio being a proxy for increased nutrient loads in reef waters [39]. In addition, *Flavobacteriaceae*-affiliated taxa were potentially diagnostic of reef ecosystem degradation, with an increasing abundance ratio of copiotrophic (e.g. OCS155, *Flavobacteraceae*, *Cryomorphaceae* and *Rhodobacteraceae*) taxa relative to oligotrophic taxa (e.g. *Pelagibacteraceae* (SAR11) and SAR86) as an index of eutrophication [39]. Finally, an increasing prevalence of opportunistic and potentially pathogenic taxa (*Rhodospirillaceae*, *Rhodobacteraceae* and *Vibrionaceae*) were also indicative of degraded inshore reef systems [39]. Another study proposed that increased *Bacteroidota* (prevalent in waters of inshore reefs in the GBR) relative to *Alphaproteobacteria* (more abundant in offshore GBR reefs) in reef surface waters could indicate enhanced macroalgae growth, elevated nutrient loads, and the start of microbial proliferation for inshore coral reefs on the GBR, which aligns with the concept of the DDAM loop [87]. Recently, a detailed overview has been provided on indicator microbes across different reef benthic habitats (i.e. nearshore and offshore, or coral- and macroalgae-dominated), highlighting the ubiquity of these patterns in the Great Barrier Reef, Caribbean, and Pacific Ocean regions (Table 1 of [76]). This baseline knowledge is relevant as it provides a list of putative indicator microbes (with their expected relative abundances in different habitats) as stable predictors of poor reef health at broad spatio-temporal scales [76], which can be used as a starting point to create applied assays for rapid reef health assessment in the field using our framework (Fig. 4).

Despite their potential, seawater microbes are still largely overlooked by reef health surveys and these seawater microbial indices are yet to be validated as useful monitoring assays in the field (Fig. 4, phase 4). Such applied research should be pursued as the seawater microbiome possesses numerous additional characteristics that align with criteria of good indicators [30], in addition to its utility to infer and predict environmental fluctuations [36, 46]. Seawater sampling and processing is simple, non-destructive and can be performed with minimal training required, which facilitates large-scale sampling alongside ongoing in situ coral health surveys that already collect metrics on water chemistry and benthic cover in the reef. Furthermore, seawater collection and processing protocols are largely standardised due to global plankton sampling expeditions such as Tara Ocean and Tara Pacific [7, 11, 14, 56–58, 65, 75] which also ensures comparability of data streams from different studies. Lastly, while monitoring whole-community dynamics is preferred compared to focusing on a subset of indicators, it is simply not feasible for macro-organisms in highly biodiverse ecosystems such as coral reefs. However, whole-community monitoring can be done when working with seawater microbial communities, and tolerance thresholds to environmental disturbances can be determined for individual microbial species from seawater. Such information could be utilised to construct cumulative species and functional sensitivity distributions, allowing to quantify the proportional impact of environmental stress on the entire microbial communities [77].

## Conclusions: a framework for incorporating microbial indicators into coral reef management

This review discusses the current state of using seawater microbes as indicators in predictive reef monitoring in the context of a five-step framework (Fig. 4), building on frameworks proposed by [64] and [111]. Many small-scale field studies combined with the emerging global studies (e.g. Tara Oceans) have identified candidate microbial taxa and genes that predict poor ecosystem and reef health [25, 31–34, 36, 39], hence the barrier does not lie in the indicator discovery phase (Fig. 4, phase 1) but largely in subsequent phases of experimental validation (Fig. 4, phase 2), formulation of microbial indices (Fig. 4, phase 3), applied research to generate microbial assays that can be used in the field (Fig. 4, phase 4), and implementation of microbial indicators in reef management and decision-making processes (Fig. 4, phase 5). Phase 1 (indicator discovery) is an ongoing process with large-scale multi-omics data streams, many that are yet to be published (e.g. Tara Pacific), fundamental to identify novel indicators and generate spatio-temporally coherent baselines of seawater microbial predictors that associate with metrics of poor reef health. Phases 2 (Experimental validation) and 3 (Formulation of seawater microbial indices to predict poor reef health) are currently a work in progress (Fig. 4) with a few studies experimentally validating microbial indicators (Fig. 4, phase 2), although primarily in the context of the DDAM loop, and we still lack conclusive experimental evidence that water-born microbial pathogens can indeed cause coral disease. Further, some indices based on seawater microbes (most notably the microbialisation scores) have been formulated to assess reef health (Fig. 4, phase 3), but these indices are still not used in standard reef monitoring.

Validated microbial-based molecular assays for rapid screening of seawater microbial indicators to predict reef decline are yet to be crafted (e.g. screening for anaerobic microbes to predict hypoxia-induced coral mortality events), hence phase 4 (applied research to craft microbial diagnostic and predictive tools) still needs to be developed. Such rapid and cost-effective assays based on seawater microbes (PCR, magnetic beads, and proteomic/colorimetric assays) have been successfully applied for environmental management (Fig. 4, phase 5), although generally inform on single stressors or have a narrow focus on risks to human health and well-being. Some examples include the presence/increased abundance of coliforms in public swimming waters indicating faecal pollution [112, 113], enrichment of antibiotic resistance genes indicating human impact [114], enrichment of hydrocarbon-degrading taxa and genes tracking oil spills [115] and anaerobic genes from sulphur-oxidising bacteria as indicators to trace the spread of oxygen minimum zones in the ocean [116]. Instead of assaying the reef environment for individual microbial indicators, it is potentially more productive to compile a list of target microbial taxa and functions that associate to poor reef health. For example, potentially pathogenic microbes increase in abundance and/or activity at elevated temperature and nutrient concentrations [85, 86, 101, 117]. Therefore screening for these taxa is a necessity during the summer period, particularly before, during and after bleaching events when coral health becomes compromised.

To move towards proactive reef management, improved communication between researchers and practitioners is needed to determine whether microbial indicators are desired in reef monitoring, as well as a cost/benefit analysis to identify which putative markers should be prioritised in applied research to develop targeted microbial-based assays. By reviewing current knowledge gaps, we highlight that seawater microbes should not be overlooked in reef monitoring efforts as marine plankton is an essential proxy of reef health, and we hope this review will catalyse further research towards predictive reef microbial monitoring and proactive management, which can be achieved if objectives are aligned between scientists, managers, and funding bodies.

## Data Availability

Not applicable.

## Abbreviations

IMOS: Australia’s Integrated Marine Observing System
GBR: Great Barrier Reef
DDAM: Disease, dissolved organic carbon, algae, microbes
DOC: Dissolved organic carbon
LDA: Linear discriminant analysis
rRNA: Ribosomal ribonucleic acid
DOM: Dissolved organic matter
